# Negative symptoms and self-reflection increase alienation in individuals at clinical high-risk for psychosis

**DOI:** 10.3389/fpsyt.2025.1560774

**Published:** 2025-09-10

**Authors:** Melina Mendonça, Natalia Mansur Haddad, Leonardo Peroni de Jesus, Julio Cesar Andrade, Feten Fekih-Romdhane, Mauricio Henriques Serpa, Martinus Theodorus van de Bilt, Wagner Farid Gattaz, Wulf Rössler, Alexandre Andrade Loch

**Affiliations:** ^1^ Laboratório de Neurociências (LIM 27), Instituto de Psiquiatria, Hospital das Clínicas Universidade de Sao Paulo, Sao Paulo, SP, Brazil; ^2^ The Tunisian Center of Early Intervention in Psychosis, Department of Psychiatry “Ibn Omrane”, Razi Hospital, Manouba, Tunisia; ^3^ Faculty of Medicine of Tunis, Tunis El Manar University, Tunis, Tunisia; ^4^ Instituto Nacional de Biomarcadores em Neuropsiquiatria (INBION), Conselho Nacional de Desenvolvimento Científico e Tecnológico, Sao Paulo, SP, Brazil; ^5^ Laboratory of Psychiatric Neuroimaging (LIM-21), Department and Institute of Psychiatry, University of Sao Paulo Medical School, Sao Paulo, Brazil; ^6^ Department of Psychiatry and Psychotherapy, Charité University of Medicine, Berlin, Germany

**Keywords:** schizophrenia, at risk mental state, attenuated psychosis, stereotype, prejudice, discrimination

## Abstract

**Objective:**

To assess stigma and cognitive insight, and their interplay with symptoms in individuals at clinical high risk for psychosis (CHR).

**Methods:**

Individuals were screened for CHR status and then assessed with the Internalized Stigma of Mental Illness, Beck Cognitive Insight Scale and Perceived Devaluation and Discrimination Scale. Spearman’s rho correlations were used to analyze data on scales and symptoms. Mediation analysis models were performed.

**Results:**

56 CHR and 18 controls were drawn from the general population. CHRs showed lower perceived discrimination and greater cognitive insight than controls. CHR individuals showed a positive correlation between alienation and both positive and negative symptoms, a positive correlation between cognitive insight and both negative and general symptoms. Strong correlations between cognitive insight and internalized stigma were observed. In the mediation analysis, alienation had an influence of negative symptoms and self-reflection. Also, alienation had the influence of negative symptoms via self-reflection.

**Conclusions:**

Our data highlights the importance of identifying pronounced negative symptoms in CHR individuals as a reinforcer of internalized stigma. In this sense, addressing stigma in CHR should carefully consider negative symptoms, given their influence on alienation both directly and indirectly via self-reflection.

## 1 Introduction

Schizophrenia spectrum disorders (SSD) are severe conditions usually associated with significant distress and impairment in broad areas of life. The estimated burden of schizophrenia in the US was $343.2 billion in 2019—which has doubled since 2013 (1). The diagnosis is frequently identified with negative stereotypes, such as dangerousness and unpredictability (2). Moreover, SSD are more severely stigmatized than other mental illnesses, such as depression and anxiety (3, 4), being among the most stigmatized conditions worldwide (5, 6). In this context, to avoid people from developing such a burdensome disorder, research efforts have been focusing on prevention and on the identification of preclinical phases of SSD.

To address early stages of SSD, the clinical high-risk for psychosis framework (CHR) was created in the 90’s (7). CHR criteria comprise three syndromes: (1) attenuated psychotic symptoms (APS); (2) brief, limited, intermittent psychotic symptoms (BIPS); and (3) presumed genetic vulnerability (schizotypal personality or family history of psychosis in a first-degree relative), associated with a decline in general functioning over the last 12 months (GRD) (8). The development of the CHR criteria was intended to prevent SSD and consequently to avoid the great burden related to them, including stigma. Up until now much has been done in the field, but there has been a growing concern that early identification of individuals in at risk conditions might expose many young people to potential stigmatization.

A recent systematic review including 38 studies observed that there is indeed stigma towards CHR individuals, mainly in the forms of perceived public stigma and public stigma (9). Data show that CHR frequently complain about status loss and discrimination. Moreover, they experience more internalized stigma and perceive more discrimination than healthy subjects or patients with non-psychotic disorders. Internalized stigma—or self-stigma—refers to the process by which individuals with mental illness endorse and apply to themselves negative stereotypes and prejudices held by society, leading to diminished self-worth, shame, and social withdrawal (10, 11). As for perceived discrimination, it refers to an individual’s subjective perception of being treated unfairly, devalued, or excluded because of their mental illness. It constitutes a core element of stigma and acts as a psychosocial stressor linked to poorer mental health outcomes, including greater symptom severity and reduced social functioning (12–14). Findings, however, are not unambiguous, as the CHR state has also been associated with positive outcomes—for instance, validation and relief (15). But in general, it has been hypothesized that individuals labeled as CHR may feel as though they have a quasi-diagnosis, with negative concepts related to SSD ‘contaminating’ the pre-clinical label (16).

In this sense, an important factor that contributes to the stigma towards CHR is the lack of understanding about the psychosis *continuum* (17). Community cohorts point to a 10-20% prevalence of at least one lifetime psychotic symptom among general population respondents (18). However, CHR prevalence is thought to be around 1.7% in the general population (19), and SSD encompass less than 0.3% of prevalence (20). This gradient of severity favors the “overspilling” of stigma from the SSD category to other less severe and non-clinical forms of psychotic manifestations along the psychosis *continuum*, such as the CHR. This is further worsened by the fact that there is still a large number of false positives generated in CHR identification (21–23).

Therefore, it worries that stereotypes usually associated with SSD would be directed towards the CHR population, causing harm to these individuals. Internalized stigma, identity problems, shame and discrimination would entail harmful consequences for the development of CHR’s personality, self-confidence, and social/academic/professional aspirations (24). Although assessing stigma in this preclinical population is essential, it is also challenging, since people tend to hide symptoms and prejudiced ideas to be seen favorably, in a process known as “social desirability bias”, which can compromise study results (25). Furthermore, the distinction between susceptibility and disease is unclear and may be even more subtle for the general public who may understand CHR as indistinguishable from schizophrenia itself (24).

In this context, the analysis of cognitive insight in CHR subjects may provide valuable information about which profiles are more or less affected by stigma. Cognitive insight refers to the capacity to reflect on and re-evaluate one’s own distorted beliefs (26). In psychotic disorders, reduced cognitive insight is often associated with strong conviction in delusional ideas and misinterpretations, limiting the individual’s ability to question experiences such as hallucinations (27). This deficit is linked to poorer treatment engagement and persistence of symptoms, whereas higher cognitive insight correlates with better awareness and functional outcomes. As such, cognitive insight can help understand the relationship between stigma and psychopathology exhibited by CHR subjects (28). Hypothetically, individuals who are not aware of the relationship between their experiences and a psychiatric condition are less likely to perceive themselves as part of a stigmatized group/condition. Hence, assessing the degree of insight and how it relates to reported atypical experiences becomes particularly relevant (29).

In the present study, we investigated stigma and cognitive insight in a CHR population, examining their relationship with clinical symptoms. Specifically, we explored whether cognitive insight is associated with internalized stigma and perceived discrimination, and how these variables interact. We hypothesized that: (1) CHR individuals would report higher levels of internalized stigma and perceived discrimination compared to healthy controls; (2) CHR individuals would exhibit impairments in cognitive insight relative to controls; and (3) greater cognitive insight would be associated with lower levels of internalized stigma and perceived discrimination.

## 2 Methods

### 2.1 Sample and procedures

This study is part of the Subclinical Symptoms and Prodromal Psychosis (SSAPP) Project, which consists of a population-based cohort study based in São Paulo City, Brazil, involving over 7500 individuals aged 18–35 years (30) For the present study, we used data from the second and third recruitment waves, from 2020 and 2022. Briefly, the second wave of recruitment was carried out during the pandemic, and the sample was recruited by a specialized research company through a random telephone number generator. A quota sampling strategy to balance possible distortions, according to age and sex based on the National Household Sample Survey, was used. For the 2022 sample, a household survey with face-to-face interviews was carried out in the general population of São Paulo, following an epidemiological design. In total, we had 4502 individuals recruited from the general population of the city of São Paulo for the cohort study.

Individuals were interviewed (either by telephone—second wave—or face to face—third wave) using the Prodromal Questionnaire-16 version (PQ-16) and the Basic Symptoms scale (BS), following previously published screening procedures (31). The PQ-16 is a shorter version of the original 92 items used in the Prodromal Questionnaire (PQ) (32), which consists of a self-report questionnaire with 16 items to screen for CHR of developing psychosis (33). The BS is a criterion based on the basic symptoms of self-experienced disturbances in perception and cognition that are present in the initial manifestations of psychosis risk (34). According to recent publications, the use of these two instruments would be effective for screening individuals with at-risk mental states (31). The recommendation is that a score of 10 or more in the sum of the PQ-16 and BS scores should be considered as a positive screening (31).

Individuals screened as positive were invited to an interview at the Institute of Psychiatry, University of Sao Paulo, Brazil. They were assessed with the Structured Interview for Psychosis Risk Syndromes (SIPS) (35, 36) for CHR status, and with the Structured Interview for DSM-5 diagnosis (SCID-5) (37). After the assessment, 82 individuals were evaluated as CHR, and 93 as healthy controls. All subjects had no psychiatric diagnosis, both CHRs and healthy controls. Further details on the study procedures can be found elsewhere (22, 30, 38, 39).

For the present study, we used data from 56 CHR and 18 random controls, who completed all the required instruments for inclusion in the work (PDD, ISMI and BCIS, see below).

Research was approved by the local and national ethics committee (National Committee on Research Ethics #1.709.439, University of Sao Paulo Ethics Committee 1.540.350, CAAE 53536816.0.0000.0065.).

### 2.2 Instruments and scoring

The SIPS (40) is a structured diagnostic interview instrument used to diagnose three prodromal syndromes for psychosis: brief intermittent psychotic symptoms syndrome (BIPS, experience of clinical psychotic symptoms that do not meet sufficient criteria for a full psychotic episode), genetic risk and deterioration syndrome (GRD, the presence of a first-degree relative with any psychotic disorder or personally meeting the Diagnostic and Statistical Manual of Mental Disorders (4th ed.; DSM-IV) criteria for schizotypal personality disorder and experiencing a drop in global functioning) and attenuated positive symptom syndrome (APS, attenuated psychotic symptoms that have not yet reached a psychotic level) (35, 36). The SIPS also includes the Schizotypal Personality Disorder Checklist (41), a family history questionnaire (42) and the Global Assessment of Functioning scale (43).

SCID-5 (Structured Clinical Interview for DSM-5, Portuguese version) was also used to identify any diagnosis compatible with the DSM-5 in participants (37, 44).

The Internalized Stigma of Mental Illness scale (ISMI) is a 29-item self-report instrument developed to assess internalized stigma in individuals with mental disorders, using a four-point Likert scale (1=strongly disagree, 2=disagree, 3=agree, 4=strongly agree). It comprises five subscales: alienation, stereotype endorsement, perceived discrimination, social withdrawal, and stigma resistance (45). The original version demonstrated strong psychometric properties, including high internal consistency (Cronbach’s alpha ranging from 0.76 to 0.90 across subscales), good test–retest reliability, and validity evidence across different diagnostic groups. The Brazilian version (ISMI-BR), validated by Ronzani et al. (46), also showed good internal consistency (α = 0.83 for the total scale) and preserved the original five-factor structure, supporting its cultural equivalence and reliability in Brazilian samples. For the present study, the ISMI-BR was adapted to reflect the experiences of individuals without a formal psychiatric diagnosis who report unusual or subclinical experiences, aligning the items to this population’s context. Mean values for each subscale were used for analysis (sum of scores divided by number of items). Higher scores indicate greater internalized stigma.

The Beck Cognitive Insight Scale (BCIS) is a self-report instrument composed of 15 items rated on a four-point Likert scale (0=strongly disagree, 1=disagree, 2=agree, 3=strongly agree), designed to assess cognitive insight via two subscales: self-reflection (9 items) and self-certainty (6 items), with a composite index calculated by subtracting mean self-certainty score from mean self-reflection score. The higher the composite index, the greater the overall cognitive insight. The original scale demonstrated satisfactory psychometric properties, including internal consistency (Cronbach’s alpha ≈ 0.67–0.70 for subscales) and a stable two-factor structure (26).

The Perceived Devaluation and Discrimination Scale (PDD) is a 12-item instrument designed to assess perceived stigma, with items rated on a four-point scale (1=strongly disagree, 2=disagree, 3=agree, 4=strongly agree). The original scale has demonstrated good psychometric properties, including high internal consistency (Cronbach’s alpha ranging from 0.78 to 0.91) and a unidimensional structure (47). A validated Portuguese version with adequate internal consistency (α = 0.71) was developed by Duarte et al. (48). For the present study, we adapted the scale to better capture the experiences of individuals with atypical symptoms, as previously done in other studies with CHR populations. Positive items had their scores reversed, and then mean score was calculated by summing up responses and dividing by 12. Higher mean scores denote more perceived stigma.

### 2.3 Statistical analysis

Statistical analysis was conducted with the Rcmdr statistical package in version 2.8-0, and SPSS version 29.0 for macOS. Categorical data were described with number and percentage. Continuous data were described with mean/median and standard deviation and were tested for normality with the Shapiro-Wilk test and Kolmogorov-Smirnov test. To determine whether there were differences between CHR and controls in terms of sociodemographic data, the Chi-square test was performed for categorical variables, the t-test for continuous variables with normal distribution versus discrete, as well as the non-parametric Wilcoxon rank sum test for continuous variables without normal distribution versus discrete variables. Differences between the two groups in the stigma, insight and SIPS scales were also assessed using the non-parametric Wilcoxon rank sum test (ordinal variables). Spearman’s rho correlations were performed to analyze relationships between each scale and the four dimensions of the SIPS. Correlations were also made between the stigma and insight scales (PDD, BCIS and ISMI). Finally, a mediation analysis was conducted to see the direct and indirect effects of symptoms and stigma on cognitive insight. This was carried out with SPSS macro-PROCESS version 4.2. As our hypothesis was that symptoms and insight might influence stigma, both with direct and indirect effects, we adopted Model 4, with 5,000 bootstrap samples and 95% percentile confidence interval. Several models were tested, with SIPS symptom dimension scores and BCIS scores entered either as mediators or independent variables, and internalized stigma (ISMI scores) as the dependent variable. The model with the best fit is displayed in the results. More complex models—such as regression models—were not used due to statistical power concerns (small sample size).

## 3 Results

### 3.1 CHR versus control subjects

Our sample consisted of 56 CHR and 18 controls. No statistical differences between CHR and controls were observed for sociodemographic variables, and for alcohol use. As expected, CHR subjects and controls had a significant difference in all SIPS subscales (all p-values<0.01) (**Table 1**).

**Table 1:** Characteristic of samples.

	CHR	Controls	p
Age (mean, SD)	29.76 (4.60)	29.11 (5.54)	0.671
Years of education (mean, SD)	7.39 (1.43)	7.33 (0.97)	0.958
Gender (female; *n*,%)	38 (67.56%)	10 (55.56%)	0.342
Employed (yes; *n*,%)	44 (78.57%)	16 (88.89%)	0.331
AUDIT (mean, SD)	4.66 (5.82)	6.61 (6.09)	0.126
SIPS positive symptoms (mean, SD)	1.29 (0.75)	0.51 (0.61)	**0.000**
SIPS negative symptoms (mean, SD)	0.96 (0.71)	0.45 (0.46)	**0.004**
SIPS disorganization symptoms (mean, SD)	0.64 (0.43)	0.35 (0.40)	**0.000**
SIPS general symptoms (mean, SD)	1.24 (0.73)	0.64 (0.59)	**0.003**
PDD (mean, SD)	4.21 (0.80)	4.56 (0.42)	**0.021**
ISMI Total score (mean, SD)	1.85 (0.35)	1.70 (0.37)	0.159
ISMI Stigma Resistance (mean, SD)	2.31 (0.63)	2.21 (0.36)	1
ISMI Social Withdrawal (mean, SD)	1.79 (0.58)	1.68 (0.55)	0.539
ISMI Alienation (mean, SD)	1.74 (0.52)	1.55 (0.48)	0.204
ISMI Stereotype Endorsement (mean, SD)	1.64 (0.47)	1.64 (0.51)	0.933
ISMI Discrimination (mean, SD)	1.77 (0.57)	1.48 (0.57)	0.087
BCIS Composite Index (mean, SD)	6.66 (4.21)	4.44 (3.90)	**0.046**
BCIS Self-reflection (mean, SD)	14.26 (4.76)	12.87 (3.90)	0.183
BCIS Self-certainty (mean, SD)	7.60 (3.60)	8.44 (3.01)	0.212

*CHR*, clinical-high risk; *AUDIT*, Alcohol Use Disorders Identification Test; *SIPS*, Structured Interview for Prodromal Syndromes; *PDD*, Perceived Devaluation and Discrimination Scale; *ISMI*, Internalized Stigma of Mental Illness; *BCIS*, Beck Cognitive Insight Scale. Bold: Statistically significant differences.

Controls scored significantly higher on the PDD compared do CHR (p=0.021). There was no significant difference between CHR and controls for ISMI subscales and their total score. In BCIS, there was a significant difference for the composite index (p=0.046), CHR scoring higher than controls (**Table 1**).

### 3.2 Correlations between stigma, insight, and symptoms among CHR individuals

Concerning internalized stigma in CHR subjects, the ISMI–alienation subdimension correlated with positive (r=0.2960, p=0.043) and negative (r=0.3236, p=0.026) symptoms (**Table 2**). There was a negative correlation between ISMI–stigma resistance and positive symptoms (r=-0.2949, p=0.044). None of these correlations survived correction for multiple comparisons.

**Table 2:** Internalized stigma versus symptoms.

	Internalized stigma of mental illness (ISMI)					
Structured Interview for Prodromal Symptoms (SIPS) dimensions	Alienation	Stereotype endorsement	Discrimination	Social withdrawal	Stigma resistance	Total score
Positive symptoms	0.2960*	-0.0697	0.0487	0.2393	-0.2949*	0.0567
Negative symptoms	0.3236*	0.1519	0.1703	0.2799	0.0081	0.2428
Disorganization symptoms	0.2509	0.0541	0.1035	0.1555	-0.0149	0.1239
General symptoms	0.1953	-0.0091	0.0538	0.2572	-0.0433	0.0971

*: p<0.05

For Cognitive Insight (BCIS) in CHR, there was a significant correlation between the Composite Index and negative symptoms (r=0.4250, p=0.003), as well as with general symptoms (r=0.3808, p=0.008) (**Table 3**). For self-reflection, there was a significant correlation for negative (r=0.2931, p=0.045), disorganization (r=0.3357, p=0.021), and general (r=0.3610, p=0.013) symptoms. All correlations, except for negative symptoms versus self-reflection, survived correction for multiple testing.

**Table 3:** Cognitive insight versus symptoms.

	Beck Cognitive Insight (BCI)		
Structured Interview for Prodromal Symptoms (SIPS) dimensions	Composite index	Self-certainty	Self-reflection
Positive Symptoms	0.1305	0.2182	0.2398
Negative Symptoms	0.4250**	0.0699	0.2931*
Disorganization	0.2186	0.1928	0.3357*
General Symptoms	0.3808**	0.1378	0.3610*

*: p<0.05, **: p<0.01, **bold**: significant after Bonferroni correction for multiple comparisons.

Perceived Discrimination: the PDD scale did not correlate with the SIPS subdimensions (data not shown).

### 3.3 Correlations between stigma and insight

In CHR, the BCIS composite index showed a significantly positive correlation with the ISMI total score (r=0.3216, p=0.027) (**Table 4**). There was also a positive correlation between the BCIS composite index and ISMI´s subdimensions of alienation (r=0.3398, p=0.019), discrimination (r=0.3278, p=0.024) and social withdrawal (r=0.2917, p= 0.047), but they did not survive Bonferroni correction.

**Table 4:** Cognitive insight versus internalized stigma.

	Beck Cognitive Insight (BCI)		
Internalized stigma of mental illness (ISMI)	Composite index	Self-certainty	Self-reflection
Alienation	0.3398*	0.3846**	0.4788**
Discrimination	0.3278*	0.3697*	0.5027**
Social Withdrawal	0.2917*	0.4982**	0.5088**
Stereotype Endorsement	0.2316	0.3816**	0.4353**
Stigma Resistance	-0.0825	-0.4874**	-0.4519**
Total	0.3216*	0.3263*	0.4234**

*=p<0.05, ** =p<0.01, **bold**: significant after Bonferroni correction for multiple comparisons.

For BCI subscales, self-certainty was related to all ISMI measures. However, only the correlations between BCI self-certainty versus ISMI social withdrawal (r=0.4982, p=0.000) and ISMI stigma resistance (r=-0.4874, p= 0.001) survived Bonferroni correction. Likewise, self-reflection was related to all ISMI measures, and all correlations survived Bonferroni correction.

There was no significant correlation between the PDD and the other scales.

### 3.4 Mediation analysis

**Figure 1:**
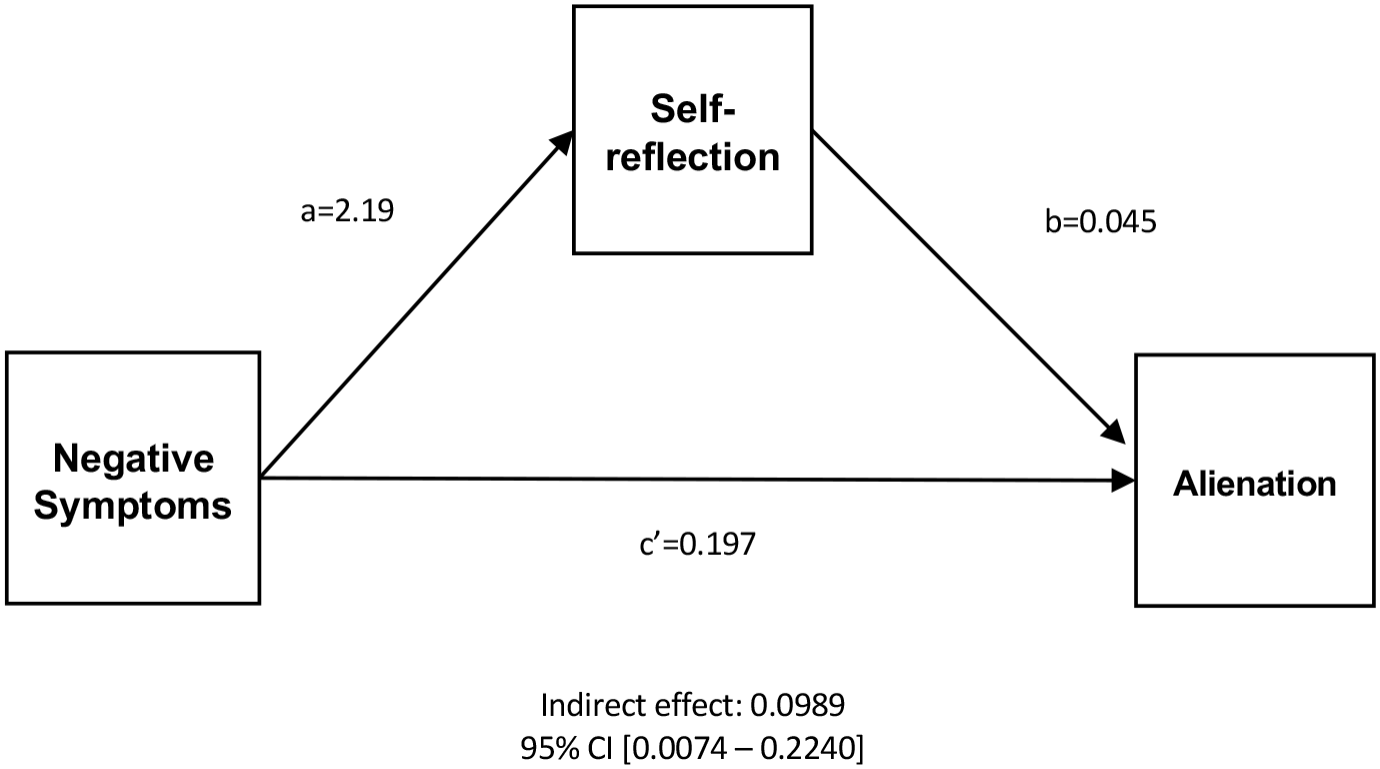
Mediation analysis.

To account for the multiple relationships, positive symptoms, negative symptoms, general symptoms, ISMI Alienation, ISMI Stigma Resistance, BCIS composite index, and BCIS self-reflection entered the mediation analysis. **Figure 1** represents the final model achieved, including only which were significantly correlated in the mediation model—i.e., SIPS negative symptoms, BCIS self-reflection, and ISMI alienation. The indirect effect of negative symptoms on alienation through self-reflection was statistically significant, with B=0.099, and a bootstrap 95% CI [0.007,0.224], based on 5000 samples. The direct effect was also significant, B=0.197, p=0.033, 95%CI [0.017 - 0.378]. This pattern indicates partial mediation, with negative symptoms associated with alienation both directly and indirectly through self-reflection. Results shown are unstandardized. Summarizing, the greater the negative symptoms, the higher the alienation, with both a direct effect and an undirect effect through increased self-reflection.

## 4 Discussion

This study set out to investigate the levels of stigma and cognitive insight in individuals at clinical high risk for psychosis (CHR), as well as how these factors relate to clinical symptoms. Our findings provide important data into the interplay between symptoms, stigma, and cognitive insight in a CHR sample. Notably, controls reported greater perceived discrimination and lower cognitive insight compared to CHR individuals. Among CHR participants, both positive and negative symptoms were positively associated with feelings of alienation, while lower positive symptoms were linked to greater stigma resistance. Negative and general symptoms were significantly related to cognitive insight, and strong correlations were observed between cognitive insight and internalized stigma. Mediation analysis revealed negative symptoms influenced alienation both directly and indirectly, via increased self-reflection. These findings clarify the complex relationships between clinical symptoms and stigma, emphasizing the central role of negative symptoms and cognitive insight in shaping internalized stigma among CHR individuals. This contributes to a better understanding of stigma mechanisms and highlights potential targets for interventions in this vulnerable population.

### 4.1 Stigma in CHR versus controls

The absence of difference between CHR and controls in the total score of the internalized stigma scale and its subdimensions is contrary to our expectation that some degree of internalized stigma would be already present in CHR individuals. Previous studies in this population show that negative thoughts and emotions about themselves are more frequent than in healthy controls, as well as greater awareness of stereotypes (15). On the other hand, most studies involved clinical populations, already monitored or seeking help, and who therefore already presented with some type of suffering or discomfort related to symptoms and disabilities in social life. We highlight that in our sample CHR were not yet identified with labels related to mental disorders, and were not seeking help. So, likewise controls, they may not have yet endorsed stereotypes currently circulating in society about people with mental disorders (49).

Furthermore, CHR individuals scored lower on the perceived discrimination scale than controls. This is contrary to our initial expectation, that CHR individuals already perceived some degree of discrimination, as reported in a systematic review by Colizzi et al. (15). The difference in our finding can be explained by the use of a different scale to measure discrimination, including conditions associated with discrimination based on gender, age, ethnicity, skin color, religion or sexual orientation (15).

On the Beck Cognitive Insight scale, CHR individuals scored higher on the composite index than controls. On the contrary, in a recent meta-analysis, cognitive insight was similar between CHR and controls (50). Another study with subjects with a diagnosis of psychosis, lower self-reflection and a higher self-certainty scores were observed, with a consequent lower composite index of cognitive insight (CI = SR - SC) (26). Again, assessing individuals in the pre-diagnostic phase, who are not yet seeking help, could explain our results.

### 4.2 Internalized stigma, cognitive insight and CHR symptoms

There was no significant association between SIPS symptoms and internalized stigma in our sample, unlike other studies, which found a significant correlation between the total ISMI score and SIPS positive (51) or negative symptoms (52). A study (53) comparing CHR and individuals with SSD found no significant differences in the total ISMI score or its subdimensions between these two groups, suggesting that internalized stigma may already be present in pre-psychotic stages. The presence of internalized stigma in pre-psychotic phases is justified by important consequences the CHR state can have on the quality of life of these individuals, as evidenced by Akouri-Shan et al. (51). In their study, total ISMI score was mediated by both SIPS positive symptoms and the subjective sensation of quality of life.

Regarding ISMI subscales, there was a trend relationship between positive and negative symptoms, and alienation, in line with the findings of other studies that also used ISMI scale (51, 52). Alienation appears to be one of the most common forms of internalized stigma experienced by people with schizophrenia and is associated with feelings of inferiority, loss of social status, as well as social exclusion due to mental illness. It has also been associated with a lower frequency of social contact among psychotic individuals and had a moderate effect on a quality of life (51). In our study, stigma resistance showed a negative trend correlation with positive symptoms. This means that the more the positive symptoms, the less the individual resists stigma and the more the individual is affected by stigmatizing attitudes (54). We could argue that this association may be the result of being more isolated from social life due subclinical to hallucinations and delusions. Nevertheless, it is important to consider that the stigma resistance subscale has not been included in the ISMI total score in several previous studies due to its relatively weak correlation to the other ISMI subscales (45).

The significant positive correlation between the BCIS composite index and negative and general symptoms, appears to result from greater self-reflection. Xu et al. (55) also observed self-reflection and the composite index to be positively correlated with negative symptoms in CHR. They suggested there might be an association between self-reflection and negative affect—such as depression and anxiety—strongly associated with negative symptoms in early onset psychosis (55–57). We could also list cultural differences to justify this pattern of greater self-reflection among individuals with more negative and general symptoms in our sample. Perhaps these symptoms are more uncomfortable and draw more attention to these individuals in our society versus other countries, since positive symptoms can to some extent be explained local religious frameworks (39).

### 4.3 Stigma and insight in CHR subjects

The moderate positive correlation between cognitive insight and internalized stigma points to a relationship in which the greater the cognitive insight, the greater the experience of internalized stigma in CHR individuals. This may suggest that the anticipation of a diagnosis of a serious mental disorder in the future is already arousing the internalization of stigmas, as in a previous study, which reported an association between cognitive insight and clinical insight in CHR individuals (58). The association between self-reflection and alienation among CHR reinforces the previous results found by Sportel et al. (53).

### 4.4 Negative symptoms and self-reflection influencing alienation

Internalized beliefs about stigma associated with CHR specific symptoms could manifest in shame, alienation and differentness, or as feelings of ‘not fitting in with others’ (59–61). In this context, the positive correlation between negative symptoms and alienation could result from the idea that perceiving oneself with negative symptoms can trigger individuals to identify with stigmatizing ideas about this condition. Likewise, alienation intensifies the withdrawal from social life and isolation. Therefore, therapies aimed at monitoring CHR individuals (62–64) could be more assertive when taking into account the relationship between negative symptoms, internalized stigma and cognitive insight.

A study found negative symptoms to impact CHR individuals’ functional outcome. It explained between 7 and 35% of the variance on measures of social- and role-functioning, self-report social functioning, and quality of life, even when controlling for the effect of antipsychotic medication, depressive symptoms, and neurocognition (65). Therefore, it would be expected that the functional impairment resulting from negative symptoms influences individuals to suffer from more social isolation—which is also present in alienation as a component of internalized stigma.

The validation study of Beck’s cognitive insight scale (2004) describes the self-reflective component as the patient’s ability and willingness to observe their mental productions and consider alternative explanations. It also represents the ability to reevaluate experiences and correct distorted beliefs and misinterpretations. However, Sportel et al. (53) observed that it is not clear whether in CHR individuals, cognitive insight as measured by the BCIS might not reflect general awareness, but also clinical insight, associated with more self-stigma. Therefore, we found that CHR individuals who are more reflective and have more negative symptoms experience more alienation from themselves and their surroundings. We hypothesize that during the pre-psychotic phase CHR individuals distance themselves from social life both due to negative symptoms, and as a result of a greater reflective activity about their inner unusual experiences. In a reference to delusional mood, as described by Jaspers: “In the less severe degrees of these states (…) patients are aware of the change, noting that they are unable to think or pay attention; and that the entire environment appears enigmatic to them; hence they plunge into perplexed astonishment (…). They feel strange, they realize that mental illness is coming and that their conscience is enormously disturbed. These feelings are accentuated into delusional anxiety” (66).

### 4.5 Strengths and limitations

To our knowledge, this is the first study in South America to report on the association between internalized stigma, cognitive insight and symptomatology in a CHR sample. We also recruited a general population sample with individuals who were not actively seeking treatment, which constitutes a further strength of our study. Risk clinics might catch individuals in later stages of the psychosis prodrome, and this may interfere with insight, for instance. Our design allowed us to assess individuals with less biases, thus. This study has some important limitations. First, the small size of the control group limited the statistical power to detect potential differences between CHR individuals and controls. Second, the cross-sectional design does not allow for causal inferences. Although no significant differences were found between groups regarding sociodemographic variables and alcohol use, the analyses were not adjusted using multivariate models, which limits our ability to account for potential confounding effects. In other words, the observed associations between stigma, cognitive insight, and symptomatology may be influenced by unmeasured or unadjusted factors. Thus, results should be interpreted with caution. Future studies with larger samples and multivariate, longitudinal approaches are needed to better clarify these relationships.

## 5 Conclusion

It is noteworthy that in CHR subjects negative symptoms, together with greater self-reflection, seemed to influence the intensity with which they experience alienation as a measure of internalized stigma. Results might have been shaped by our Latino cultural background, which more easily contextualizes positive symptoms rather than negative symptoms. We highlight the importance of understanding the interplay between symptoms, stigma and sociocultural background in CHR individuals.

In that regard, interventions can benefit from the patient’s willingness to self-reflect to challenge stigmatizing ideas to reduce internalized stigma. Likewise, social skills training aimed at increasing social interaction can remedy the impact that negative symptoms have on alienation as internalized stigma. Finally, campaigns aimed at informing patients, family members and health professionals about risk syndromes for psychosis must consider cultural characteristics of the community, placing more emphasis on negative symptoms in societies where these symptoms may be related to a greater burden of stigma.

We also suggest that there might be important differences in level of stigma according to the different stages of psychosis, and in the interplay between symptoms and stigma. At last, we underline the need to conduct studies in samples with different sociocultural backgrounds, to understand which mechanisms of stigma are culture-specific, and which are not.

## Data Availability

The raw data supporting the conclusions of this article will be made available by the authors, without undue reservation.
